# Review of the millipede genus *Sellanucheza* Enghoff, Golovatch & Nguyen, 2004, with descriptions of three new species from Laos and redescription of *S. variata* (Attems, 1953) (Diplopoda, Polydesmida, Paradoxosomatidae)

**DOI:** 10.3897/zookeys.1282.186746

**Published:** 2026-06-23

**Authors:** Natdanai Likhitrakarn, Sergei I. Golovatch, Josiane Lips, Somsak Panha, Chirasak Sutcharit

**Affiliations:** 1 Program of Agriculture, Faculty of Agricultural Production, Maejo University, Chiang Mai 50290, Thailand Animal Systematics Research Unit, Department of Biology, Faculty of Science, Chulalongkorn University Bangkok Thailand; 2 A. N. Severtsov Institute of Ecology and Evolution, Russian Academy of Sciences, Leninsky pr. 33, Moscow 119071, Russia Program of Agriculture, Faculty of Agricultural Production, Maejo University Chiang Mai Thailand; 3 Fédération française de Spéléologie, Commission scientifique, Groupe d'Étude de Biospéologie, Lyon, France Academy of Science, The Royal Society of Thailand Bangkok Thailand; 4 Animal Systematics Research Unit, Department of Biology, Faculty of Science, Chulalongkorn University, Bangkok 10330, Thailand A. N. Severtsov Institute of Ecology and Evolution, Russian Academy of Sciences Moscow Russia; 5 Academy of Science, The Royal Society of Thailand, Bangkok 10300, Thailand Fédération française de Spéléologie, Commission scientifique, Groupe d’Étude de Biospéologie Lyon France

**Keywords:** Biodiversity, cave, endemism, key, limestone Indochina

## Abstract

The flat-backed millipede genus *Sellanucheza* Enghoff, Golovatch & Nguyen, 2004 is recorded from Laos for the first time, with three new species described from limestone caves in Khammouane and Bolikhamsay provinces, northeastern Laos: *Sellanucheza laotica***sp. nov**., *S. longispina***sp. nov**., and *S. ancorata***sp. nov**. These discoveries significantly broaden the morphological concept of the genus, necessitating an amended diagnosis to accommodate unique characters such as the presence of sternal cones on both rings 5 and 6 in *S. longispina***sp. nov**. and a highly characteristic, anchor-shaped (trifid) solenophore tip in *S. ancorata***sp. nov**. *Sellanucheza laotica***sp. nov**. shows distinct troglomorphic traits, including a small and pallid body, suggesting adaptations to a strictly subterranean existence. In addition, *S. variata* (Attems, 1953), a poorly known congener from northern Vietnam, is redescribed and illustrated based on type material from the Naturhistorisches Museum Wien, Austria. An updated identification key to all nine accepted species of the genus is provided, with a distribution map. These findings represent a major range extension for the genus into the central Indochinese Peninsula, bridging its previously known distribution between southern China and northern Vietnam. With the addition of the above three new taxa, the documented millipede fauna of Laos is updated to comprise 98 accepted species belonging to 33 genera, 12 families, and eight orders.

## Introduction

Laos is a mountainous country in Indochina, widely recognized for its significant biodiversity, particularly associated with its relatively intact tropical forests and complex limestone karst systems (Kemp 2011). Despite this richness, knowledge of the millipede fauna (Diplopoda) of Laos remains modest compared to that of the neighboring countries such as Thailand or Vietnam (Likhitrakarn et al. 2023a; Nguyen et al. 2025). A comprehensive checklist published a decade ago documented only 34 species (Likhitrakarn et al. 2014a). Since then, another 62 new species have been added (Golovatch and VandenSpiegel 2017; Jiang et al. 2017; Likhitrakarn et al. 2017, 2023b, 2024, 2025a, 2025b, 2026; Golovatch 2018, 2019; Nguyen and Sierwald 2019; Wesener 2019; Nguyen et al. 2023; Srisonchai et al. 2023a, 2023b; Wesener et al. 2023; Srikampha et al. 2025). However, this number still represents an incomplete inventory of the country’s actual millipede diversity, which has been estimated to exceed 130 species (Likhitrakarn et al. 2017). Although recent extensive surveys have gradually expanded this list, numerous genera common in the region remain unrecorded or understudied in Laos.

In the family Paradoxosomatidae, *Sellanucheza* Enghoff, Golovatch & Nguyen, 2004, was proposed as a replacement name for *Szechuanella* Hoffman, 1961, the latter being preoccupied by a genus of Cambrian trilobites (Enghoff et al. 2004). Initially assigned to the tribe Tonkinosomatini, *Sellanucheza* was subsequently transferred to the tribe Sulciferini, as the Tonkinosomatini was deemed a redundant taxon following the reassignment of its genera (Golovatch 2013a; Nguyen and Sierwald 2013). To date, *Sellanucheza* comprises only six formally described species: *S. tenebra* (Hoffman, 1961), *S. variata* (Attems, 1953), *S. grandis* (Golovatch, 1984), *S. hoffmani* Nguyen, 2011, *S. jaegeri* Golovatch, 2013, and *S. typica* Golovatch, 2013. All currently known congeners are restricted to southern China, as well as northern and central Vietnam. Therefore, the discoveries of the new species from Laos represent a significant range extension for the genus into the central part of the Indochinese Peninsula (Fig. 16).

The present paper contributes to filling in this gap by describing three new species of *Sellanucheza* discovered in limestone caves in northeastern Laos, marking the first record of the genus from the country. In addition, we provide a detailed redescription and new illustrations of *Sellanucheza variata* (Attems, 1953), a species originally described from Sapa, Vietnam (Attems 1953). This redescription is based on the re-examination of type material from the Naturhistorisches Museum Wien (NHMW), Austria, allowing for the redescription of this poorly known taxon. These discoveries not only enhance our understanding of paradoxosomatid diversity, but they also underscore the high degree of short-range endemism characteristic of the cavernicolous millipede fauna of Southeast Asia.

## Materials and methods

The present study is based on newly collected material obtained during several biological and speleological expeditions in Laos, conducted in 2016 by Explo Laos and the “Fédération Française de Spéléologie, Commission Scientifique, Groupe d’Étude de Biospéologie, France”, led by Josiane Lips, as well as by Peter Jäger and his team from the Senckenberg Research Institute, Frankfurt am Main, Germany.

All specimens were euthanized according to the American Veterinary Medical Association’s guidelines for animal euthanasia (American Veterinary Medical Association 2020). Subsequently, the material was preserved in 70% ethanol for morphological examination. The Animal Care and Use Protocol Review No. 1723018 was applied. In addition, type specimens of *Sellanucheza variata* (Attems, 1953) were examined from the collection of the Naturhistorisches Museum Wien (NHMW), Austria, allowing for a comprehensive redescription and updated illustrations to be presented.

The examined material, including the holotypes and paratypes of the new species, is deposited in the Museum of Zoology, Chulalongkorn University (**CUMZ**), Bangkok, Thailand. However, all material provided by P. Jäger has been returned, for permanent storage, to the Senckenberg Research Institute (**SMF**), Frankfurt am Main, Germany.

Live specimens were photographed in the field or laboratory using a Nikon D700 digital camera fitted with a Nikon AF-S VR 105 mm macro lens. Laboratory examinations were carried out using a Leica M205 MCA microscope. All measurements and observations of somatic and gonopodal characters were recorded using digital calipers and an eyepiece micrometer. Digital images of the specimens were captured with a Leica DMC 6200 camera and processed using the LAS automontage software to create high-depth-of-field composite images.

Detailed line drawings of gonopodal structures were prepared based on direct microscopic observations and digital photographs. For scanning electron microscopy (SEM), dissected gonopods were mounted on aluminum stubs and coated with an 8 nm gold layer using a CCU-010 high-vacuum sputter coater. SEM micrographs were obtained using a TESCAN VEGA3 scanning electron microscope operating at an acceleration voltage of 5 keV. Following SEM examination, the gonopods were returned to alcohol.

In the taxonomic catalogue sections, a letter system is applied to denote the status of taxonomic investigation: **D** stands for the original description and subsequent descriptive notes; **K** for the appearance in a key; **L** for the appearance in a species list; **M** for a mention; and **R** for new subsequent records. The terminology for denoting the gonopodal and somatic structures primarily follows Golovatch (2016), Likhitrakarn et al. (2022, 2025b) and Ng et al. (2025). The abbreviations used for specific gonopodal structures are as follows:

**pa** process A,

**pb** process B,

**fe** femorite,

**pfe** prefemoral part,

**sl** solenomere,

**sph** solenophore.

Collection localities were pinpointed using GPS (WGS84 datum), and their accuracy was further verified through Google Earth Pro version 7.3.6.

### Taxonomy

**Order Polydesmida**

**Family Paradoxosomatidae Daday, 1889**

**Tribe Sulciferini Attems, 1898**

#### Genus Sellanucheza

Taxon classificationAnimaliaPolydesmidaParadoxosomatidae

Enghoff, Golovatch & Nguyen, 2014

0BED9F91-B91D-5AEC-BD9D-F7BC976231AC

Szechuanella Hoffman 1961: 533 (D), preoccupied by Szechuanella Lu, 1959.
Szechuanella –Hoffman 1963: 586 (M, K); Jeekel 1968: 62 (M, R); Golovatch 1984: 57 (M); Shelley et al. 2000: 132 (M); Sierwald 2009: 117 (L).
Sellanucheza Enghoff et al. 2004: 39 (M, L), nomen novum.
Cemsunguria Özdikmen 2007: 433 (M), junior objective synonym (synonymized by Nguyen 2011).
Sellanucheza –Golovatch 2013a: 22 (M, K); 2013b: 330 (M, K); Nguyen and Sierwald 2013: 1295 (M, L).

##### Diagnosis

**(amended after Hoffman 1961 and Nguyen 2011)**. *Sellanucheza* is a member of the tribe Sulciferini and can be distinguished from all other genera in the tribe by the following combination of gonopodal characters: (1) telopodite provided with two or three large postfemoral processes; (2) both lamina medialis and lamina lateralis being well-developed; (3) lack of a clear demarcation between the femorite and the postfemoral region; and (4) solenophore distinctly curved ventrad to form a circular or subcircular structure together with the femorite, and bearing one or two basal processes.

##### General morphological characters.

A genus of medium to large-sized Paradoxosomatidae (body length ca 11–59 mm) with 20 rings and a normal pore formula. Paraterga variable, ranging from vestigial to strongly developed on midbody rings. Coloration typically dark brown to blackish but also including uniform light yellowish to pallid forms. Transverse sulcus usually distinct on metaterga. Sternal modifications in males always involving a large sternal lobe between coxae 4 on ring 5; additional modifications may include transverse lobes or paramedian cones on both rings 5 and 6, or well-developed ventroposterior tubercles near the coxae starting from ring 6 onwards. Male legs without adenostyles; tarsal brushes present, sometimes extending as far as ring 17. Epiproct long and flattened, with a pair of distinct lateral dentiform tubercles.

Gonopods relatively simple; coxite long, slender and cylindrical, with a dense group of setae distodorsally. Prefemoral part (pfe) short and densely setose, ca 1/3–1/5 length of acropodite. Femorite (fe) long and slender or relatively stout and curved, often with a distinct mesal groove. Distal part of femorite or base of solenophore (sph) supplied with two distinct processes: process pa typically long, spiniform, slender and acute, often with its tip curved ventrad, although sometimes short; process pb usually shorter, varying from a short acute spine or a long projection to a small or large lamina. Solenophore relatively long, coiled or curved to sheath the usually long and flagelliform solenomere (sl); distal margin of solenophore highly variable, ranging from truncate and subquadrate to deeply bifid or uniquely anchor-shaped (trifid).

##### Type species.

*Szechuanella tenebra* Hoffman, 1961, by original designation.

##### Other species included.

*Sellanucheza tenebra* (Hoffman, 1961), *S. variata* (Attems, 1953), *S. grandis* (Golovatch, 1984), *S. hoffmani* Nguyen, 2011, *S. jaegeri* Golovatch, 2013, *S. typica* Golovatch, 2013, *S. laotica* sp. nov., *S. longispina* sp. nov., *S. ancorata* sp. nov.

##### Remarks.

The genus name *Sellanucheza* was established by Enghoff et al. (2004) as a replacement name for the preoccupied *Szechuanella* Hoffman, 1961 (non Lu, 1959). Subsequently, *Cemsunguria* was proposed as an unnecessary replacement name by Özdikmen (2007), making it an instantaneous junior objective synonym of *Sellanucheza* under Article 23.1 of the ICZN (Nguyen 2011; Nguyen and Sierwald 2013). With the addition of three new species described in this study, the genus *Sellanucheza* now comprises nine formally described species. These new discoveries in the limestone karst systems of Khammouane and Bolikhamsay provinces, northeastern Laos (Fig. 16), represent a significant range extension for the genus, which was previously known only from southern China and northern and central Vietnam (Hoffman 1961; Golovatch 1984, 2013a; Nguyen 2011).

#### Sellanucheza tenebra

Taxon classificationAnimaliaPolydesmidaParadoxosomatidae

(Hoffman, 1961)

3517C922-DC8D-5542-9A18-67D30F606D3E

Fig. 1

Szechuanella tenebra Hoffman 1961: 535 (D).
Szechuanella tenebra –Hoffman 1963: 586 (M, K); Jeekel 1968: 75 (M); Wang and Mauriès 1996: 87 (L); Sierwald 2009: 125 (M).
Sellanucheza tenebra –Enghoff et al. 2004: 39 (M); Nguyen and Sierwald 2013: 1296 (L); Golovatch 2013a: 22 (M, K); 2013b: 330 (M, K).

##### Distribution.

China, Sichuan Province, Wushan, 31.07°N, 109.52°E (Hoffman 1961).

##### Remarks.

**Figure 1.**
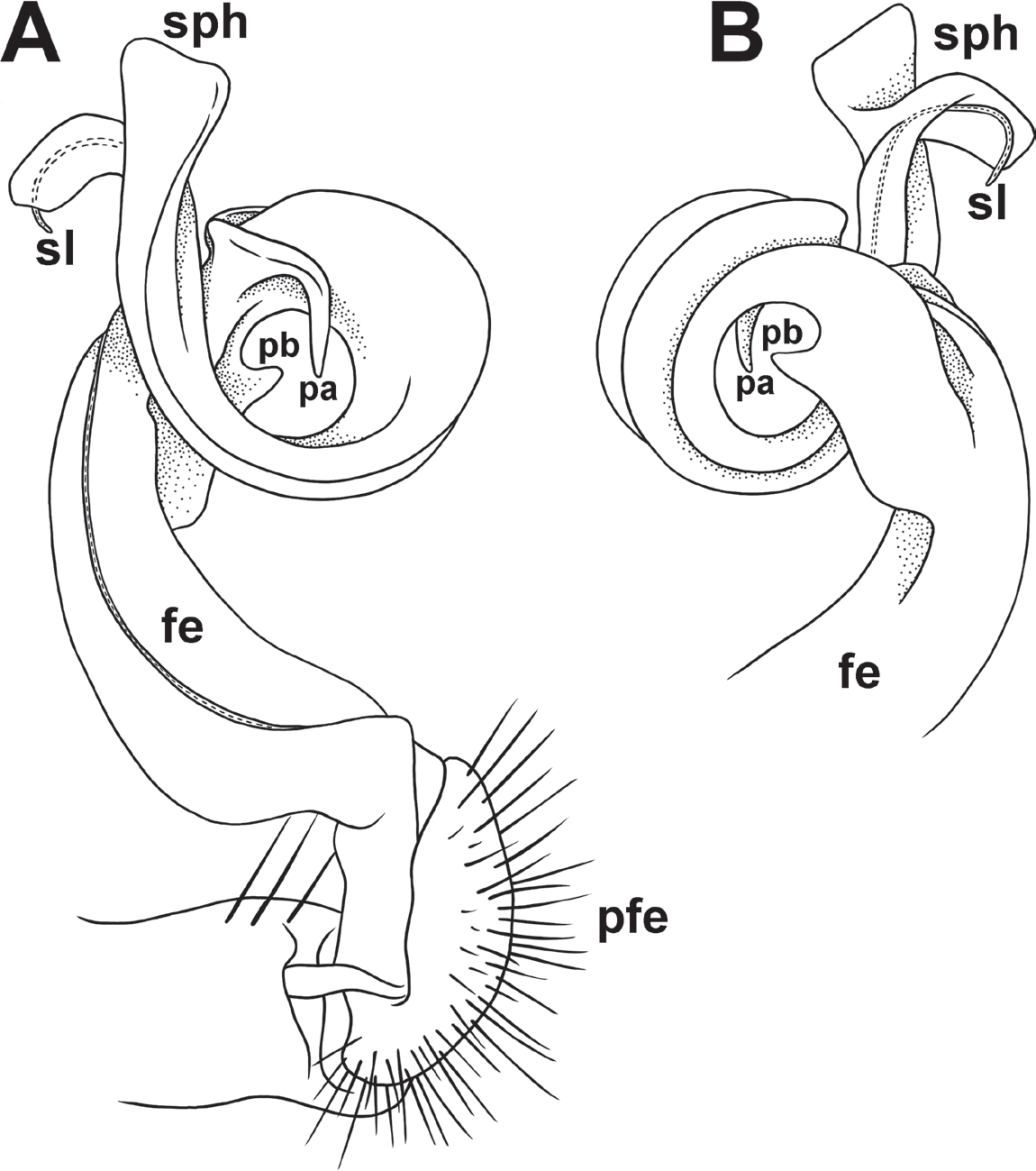
*Sellanucheza tenebra* (Hoffman, 1961). **A**. Right gonopod, mesal view; **B**. Distal part, lateral view. Drawings modified after Hoffman (1961), not to scale. Abbreviations: pa = process A, pb = process B, fe = femorite, pfe = prefemoral part, sl = solenomere, sph = solenophore.

*Sellanucheza tenebra* was originally described as the type species of the genus *Szechuanella* Hoffman, 1961, based on a male holotype from Wushan, Sichuan Province, China (Hoffman 1961). However, as the name *Szechuanella* Hoffman, 1961 was found to be preoccupied by a trilobite genus (Zhang and Fan 1960), Enghoff et al. (2004) proposed *Sellanucheza* as a replacement name. Somatically, this species is unique among congeners due to the strongly reduced paraterga on rings 3–19, these being represented only by very faint bulges devoid of any delimiting sulci. This character makes *S. tenebra* clearly distinguished from all other known species, in which the paraterga, even if poorly developed, are always demarcated by at least a dorsal sulcus. In addition, male sternum 5 is characteristic in bearing a large, hemispherical lobe between coxae 4. Regarding the gonopod, *S. tenebra* is characterized by a long, strongly coiled solenophore (sph) which is subequal in length to a subunciform femorite (fe) (Fig. 1A). Most notably, the gonopod bears a conspicuous, long and acute process pa directed ventrad, as well as a small, acute, basal process pb (Fig. 1A, B). The solenophore tip is bifid, consisting of two large, rounded, subequal, hyaline, spatulate lobules (Fig. 1A, B). These features make *S. tenebra* clearly distinguished from congeners such as *S. variata* or *S. grandis*, which typically show a relatively shorter or differently shaped solenophore. The species remains known only from the type locality in Sichuan Province, China.

#### Sellanucheza variata

Taxon classificationAnimaliaPolydesmidaParadoxosomatidae

(Attems, 1953)

0BF4A978-E100-5588-9F4A-1645F4D92422

Figs 2, 3

Nedyopus variatus Attems 1953: 170 (D).
Szechuanella variata –Hoffman 1963: 586 (D, K); Jeekel 1968: 62 (M); Golovatch 1983: 182 (M).
Sellanucheza variata –Enghoff et al. 2004: 39 (L); Golovatch 2013a: 22 (M); 2013b: 330 (M, K); Nguyen and Sierwald 2013: 1296 (L).

##### Type material examined.

• ***Syntypes*** 1 ♂, 1 ♀ (NHMW-3536), Vietnam, Laocai Province, Cha Pa (= Sapa), 1938–1939, leg. C. Dawydoff.

##### Diagnosis.

Among all known congeners, *Sellanucheza variata* appears to be most similar to *S. typica* Golovatch, 2013, but can be distinguished by the following combination of characters. Somatically, it differs from *S. typica* by the presence of pleurosternal carinae until ring 5 (vs absent in *S. typica*) and male tarsal brushes extending until ring 16 (vs ring 12 in *S. typica*). Regarding the gonopods, the femorite is notably stout (vs slender and elongate in *S. typica*). The solenophore is clearly curved and subequal in length to the femorite. Process pa is prominent, long, slender, and acute, while process pb is short, spiniform, and curved upwards.

##### Redescription.

Length ca 37 (♂) or 46 mm (♀), width of midbody pro- and metazona 2.8 and 3.3 mm (♂) or 4.6 and 5 mm (♀), respectively.

**Figure 2.**
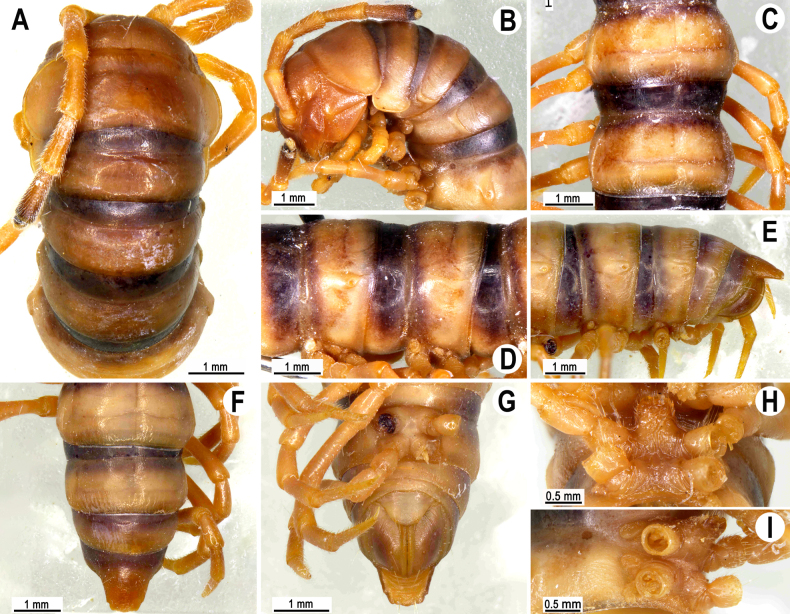
*Sellanucheza variata* (Attems, 1953), ♂ syntype. **A, B**. Anterior part of body, dorsal and lateral views, respectively; **C, D**. Rings 10 and 11, dorsal and lateral views, respectively; **E–G**. Posterior part of body, lateral, dorsal and ventral views, respectively; **H, I**. Sternal cones between coxae 4, subposterior and sublateral views, respectively.

Coloration of alcohol material after 87 years of preservation, faded to reddish brown or black-brown (Fig. 2A–F), posterior halves of metaterga, collum, paraterga and epiproct pale light brown to yellowish (Fig. 2A–F), head and antennae yellow-brown, tip of antenna dark brown (Fig. 2A), venter and legs pale brown to yellowish (Fig. 2B–I).

Clypeolabral region and vertex sparsely setose, epicranial suture distinct. Antennae moderately long (Fig. 2A, B), reaching body ring 3 (♂, ♀) when stretched dorsally. In width, head < ring 3 = 4 < 2 < collum < ring 5 < 6–17 (♂); thereafter body gently and gradually tapering.

Collum semi-lunar, with an untraceable pattern of setation, surface rugulose, middle slightly flattened; paraterga rounded subtriangular, declined ventrad, devoid of lateral incisions; posterior corner very narrowly rounded, not extending past tergal margin (Fig. 2A, B).

Tegument smooth and shining, prozona finely shagreened, metaterga finely leathery and faintly rugulose; surface below paraterga finely microgranulate (Fig. 2A–F). Postcollum metaterga with two transverse rows of setae traceable at least as insertion points when setae broken off: 3+3 in anterior (pre-sulcus) and 4+4 in posterior (post-sulcus) row, the latter being barely traceable as insertion points (Fig. 2A–F). Axial line visible both on pro- and metazona.

Paraterga well developed (Fig. 2A, C, F), lying low (at 1/2 of body), slightly upturned, but lying below dorsum; anterior edge broadly rounded and narrowly bordered, fused to callus; lateral edge without incisions; posterior corner very narrowly rounded, not produced past rear tergal margin except for ring 2; posterior edge nearly straight. Paraterga 2 broad, anterior edge angular and rounded, lateral edge without incisions (Fig. 2A, B). Calluses on paraterga narrow, demarcated by a sulcus only dorsally. Ozopore evident, lateral, lying in an ovoid groove at ca 1/3 metaterga in front of posterior corner (Fig. 2B, D, E).

Transverse sulcus distinct (Fig. 2A, C, F), slightly incomplete on metaterga 4, complete on metaterga 5–18, narrow, line-shaped, shallow, reaching the bases of paraterga, faintly beaded at bottom. Stricture between pro- and metazona narrow, beaded at bottom down to base of paraterga (Fig. 2B–F).

Pleurosternal carinae complete high crests with a sharp posterior tooth on each of rings 2 and 3, thereafter remaining visible only as a bulge anteriorly until ring 5 (Fig. 2B, D, E).

Epiproct (Fig. 2E, F) conical, rounded dorsoventrally, with two small apical papillae; tip subtrapeziform; pre-apical papillae evident, lying close to tip. Hypoproct nearly semi-circular (Fig. 2G), posterior tip broadly rounded, setiferous knobs at posterior edge very small and moderately well separated.

Sterna sparsely setose, without modifications (Fig. 2G); an entire, large, rounded, linguiform, setose, sternal lobe between ♂ coxae 4 (Fig. 2H, I). A paramedian pair of small, but evident tubercles in front of gonopod aperture. Legs moderately long and slender, midbody legs ca 1.2–1.4 (♂) or 1.1–1.3 × (♀) as long as body height, ♂ prefemora without modifications, tarsal brushes present until ♂ legs 16.

**Figure 3.**
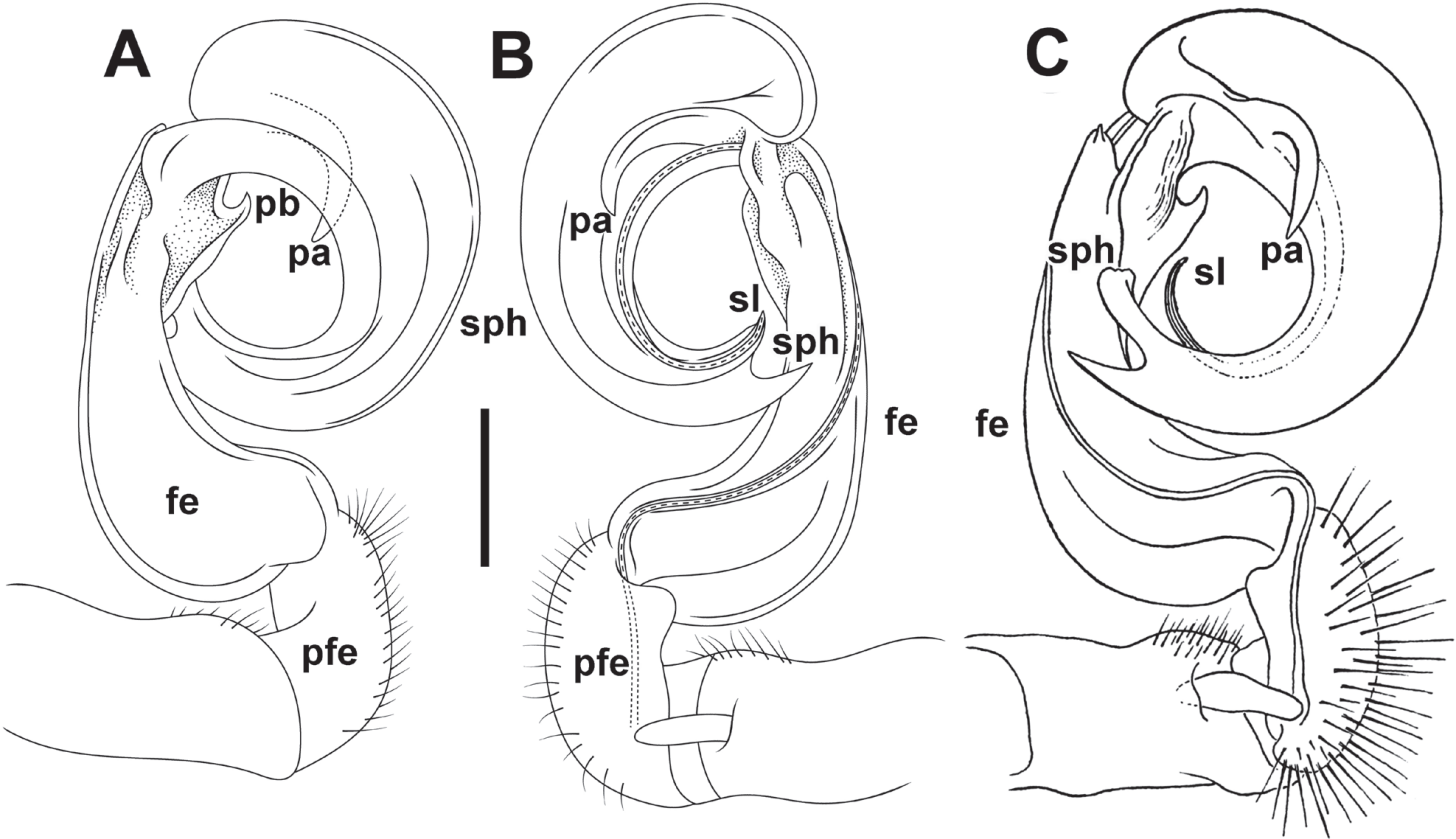
*Sellanucheza variata* (Attems, 1953), ♂ syntype. **A, B**. New drawings of right gonopod, lateral and mesal views, respectively; **C**. Left gonopod, mesal view, modified and redrawn from Hoffman (1963). Abbreviations: pa = process A, pb = process B, fe = femorite, pfe = prefemoral part, sl = solenomere, sph = solenophore. Scale bars: 0.5 mm (**A, B**).

Gonopods (Fig. 3) simple. Coxite long, slender, cylindrical, slightly curved posteriorly, densely setose distodorsally. Prefemorite (pfe) densely setose, as usual, ovoid, elongate, ca 1/3–1/4 length of acropodite (Fig. 3A–C). Femorite (fe) relatively stout, expanded basally, narrowed distad, evidently curved (Fig. 3A–C), showing a distinct mesal groove; process pb short, spiniform, slightly curved upwards, located apicoventrally (Fig. 3A, C). Solenophore (sph) large, broad, lamellar, strongly coiled, briefly bifid at tip, consisting of a straight, acute tooth and a rounded lobe with a faintly serrate margin (Fig. 3C); base of solenophore with a most prominent, long, slender, curved and acute process pa projecting mesad (Fig. 3A–C). Solenomere (sl) long, flagelliform, originating from femorite, almost fully sheathed by solenophore, with only its tip slightly exposed (Fig. 3B, C).

##### Remarks.

Although Attems (1953) and Hoffman (1963) provided descriptions of this species, *Sellanucheza variata* has hitherto remained poorly defined, mainly due to the lack of detailed gonopod illustrations. The present study provides new and detailed drawings of the right gonopod (Fig. 3A, B) based on the re-examination of the type material, complemented by a modified illustration of the left gonopod (Fig. 3C) from Hoffman (1963) to provide a complete diagnostic overview. The present redescription, based on the syntypes, serves to complete the documentation and stabilize the diagnostic features of the species. In accordance with Hoffman (1963), the type locality is confirmed as being restricted to Chapa (= Sa Pa), Lao Cai Province, northern Vietnam.

#### Sellanucheza grandis

Taxon classificationAnimaliaPolydesmidaParadoxosomatidae

(Golovatch, 1984)

85D53879-8BDE-5EA9-93A7-15B3383699B5

Fig. 4

Szechuanella grandis Golovatch 1983: 182 (nomen nudum).
Szechuanella grandis Golovatch 1984: 56 (D); Korsós and Golovatch 1989: 212 (R).
Sellanucheza grandis –Enghoff et al. 2004: 39 (L, R); Nguyen 2011: 59 (D, R); Golovatch 2013a: 22 (M, K); 2013b: 330 (M, K); Nguyen and Sierwald 2013: 1296 (L).

##### Material examined.

3♂, 4♀, Vietnam, Ninh binh, Cuc Phuong National Park, 300 m a.s.l., 20°19'4"N, 105°36'30"E, 26.07.2006, leg. S. Panha and C. Sutcharit.

##### Descriptive notes.

**Figure 4.**
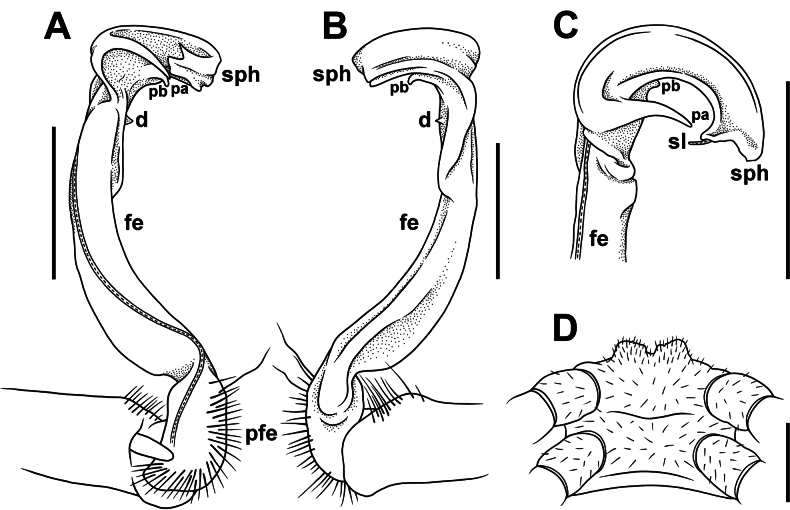
*Sellanucheza grandis* (Golovatch, 1984), ♂ new collected specimen. **A, B**. Left gonopod, mesal and lateral views, respectively; **C**. Distal part, submesal view. Abbreviations: pa = process A, pb = process B, fe = femorite, pfe = prefemoral part, sl = solenomere, sph = solenophore. Scale bars: 1.0 mm.

Length 54.3–56.2 (♂) or 51.8–57.3 mm (♀), width of midbody pro- and metazonae 4.8–5.2 and 5.7–6.1 mm (♂) or 5.6–6.1 and 6.7–7.0 mm (♀), respectively. Antennae moderately long, reaching body ring 5 (♂) or 4 (♀) when stretched dorsally. Sterna sparsely setose, shining, shallow cross-impressions, without modifications; a large, central, slightly bifid, setose lobe between ♂ coxae 4 (Fig. 4D). Legs moderately long and slender, midbody legs ca 1.1–1.4 (♂) or 1.0–1.2 × (♀) as long as body height; ♂ prefemora without modifications; tarsal brushes present until ♂ ring 11.

##### Distribution.

Vietnam, Ninh Binh, Cuc Phuong National Park (Golovatch 1984; Korsós and Golovatch 1989; Enghoff et al. 2004; Nguyen 2011); Ha Tinh Province, Huong Son District, Son Kim commune, secondary forest; HaTinh Province, Huong Son District, Cau Treo, secondary forest; Phu Tho Province, Xuan Son National Park, forest, 500 m a.s.l.; Nghe An Province, Pu Mat National Park, Khe Thoi, closed forest, near stream, 18°58'17.4"N, 104°48'20.9"E (Nguyen 2011).

##### Remarks.

Originally described as *Szechuanella grandis* from a limestone hill in Cuc Phuong National Park, Ninh Binh Province, Vietnam (Golovatch 1984), this species was transferred to *Sellanucheza* by Enghoff et al. (2004). The name *Szechuanella grandis* was first introduced as a *nomen nudum* by Golovatch (1983), as it appeared in a species list without an accompanying description or diagnosis. The species was subsequently validated and formally described by Golovatch (1984). Somatically, *S. grandis* differs clearly from congeners, including *S. tenebra* and *S. variata*, by the considerably larger body size. In contrast to *S. tenebra*, where paraterga are absent from rings 3–19, *S. grandis* retains abbreviated paraterga demarcated by distinct dorsal sulci.

The gonopods are highly distinctive, the solenophore (sph) being conspicuously shorter, at most half as long as the strongly coiled femorite (fe) (Fig. 4A, B). This character immediately separates *S. grandis* from both *S. variata* and *S. tenebra*, in which the solenophore and femorite are subequal in length (Figs 11A, 3A–C). The femorite is relatively straight, being only slightly curved (Fig. 4A, B). The postfemoral part is supplied with a small, dentiform, sublateral spine (d) (Fig. 4A, B), a large and pointed process pb (Fig. 4A–C), as well as a large, acute, mesal process pa (Fig. 4A, C). In addition, the solenophore is short, coiled, the tip being only faintly bifid and consisting of small, rounded, apical lobules (Fig. 4A, C).

The new material reported above fully agrees with the original description (Golovatch 1984), showing only slight variations in standard measurements (see “Descriptive notes” above). In contrast to other congeners which appear to be highly localized, *S. grandis* seems to be relatively widespread, the maximum distance between the known localities amounting to ca 300 km. Although the distribution range has recently been extended from the type locality in Ninh Binh Province to also cover Ha Tinh, Phu Tho, and Nghe An Provinces, the species still remains endemic to northern Vietnam.

#### Sellanucheza hoffmani

Taxon classificationAnimaliaPolydesmidaParadoxosomatidae

Nguyen, 2011

4C474559-951C-5EA5-9869-5A1577B70CA9

Fig. 5A–C

Sellanucheza hoffmani Nguyen 2011: 59 (D, K).
Sellanucheza hoffmani – Golovatch 2013b: 330 (M, K); Nguyen and Sierwald 2013: 1296 (L).

##### Distribution.

Vietnam, Kon Tum Province, Loxo Pass, ca 80 km north of Kontum, secondary forest, 800 m a.s.l.; Quang Binh Province, Minh Hoa District, Thuong Hoa commune, Phong Nha-Ke Bang region (Nguyen 2011).

##### Remarks.

**Figure 5.**
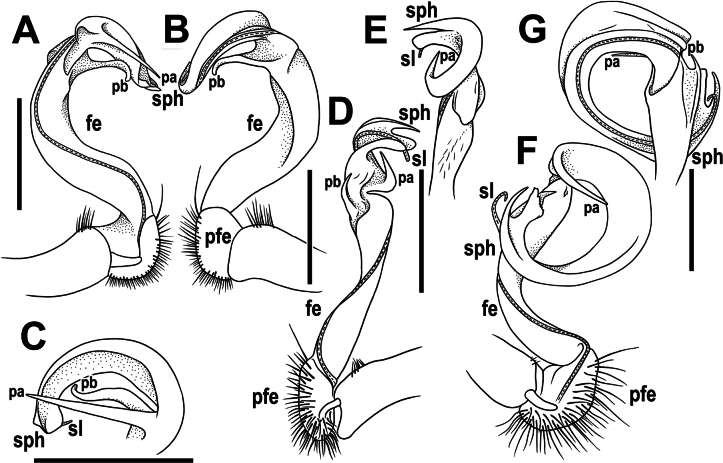
Gonopods of *Sellanucheza* species. **A–C**. *Sellanucheza hoffmani* Nguyen, 2011, ♂ holotype, left gonopod; **A, B**. Mesal and lateral views, respectively; **C**. Distal part, dorsal view; **D, E**. *Sellanucheza jaegeri* Golovatch, 2013, ♂ paratype, right gonopod; **D**. Mesal view; **E**. Distal part, lateral view; **F, G**. *Sellanucheza typica* Golovatch, 2013, ♂ holotype, left gonopod; **F**. Mesal view; **G**. Distal part, lateral view. Drawings modified after Nguyen (2011) (**A–C**) and Golovatch (2013a, 2013b) (**D–G**). Abbreviations: pa = process A, pb = process B, fe = femorite, pfe = prefemoral part, sl = solenomere, sph = solenophore. Scale bars: 1.0 mm (**A–C**); 0.5 mm (**D–G**).

This species is currently known only from central Vietnam. Somatically, this species is one of the largest members of the genus (length 52–59 mm, width 5.3–6.4 mm), being comparable in size only to *S. grandis*. However, it is readily distinguished from all congeners by male sternum 5 showing two separate, small, setose cones between coxae 4, and by the paraterga being small, but complete, represented by keel-shaped ridges demarcated by distinct sulci both dorsally and ventrally. The gonopods are particularly distinctive, the femorite (fe) being long, marginally expanded distad, with the distal part being simultaneously twisted and curved ventrad (Fig. 5A, B); the postfemoral region bears a long, basal, laminiform process pb (Fig. 5A–C); and the solenophore (sph) is supplied with a very long, nearly straight, spiniform process pa at the base of the lamina medialis (Fig. 5A, C).

#### Sellanucheza jaegeri

Taxon classificationAnimaliaPolydesmidaParadoxosomatidae

Golovatch, 2013

9EC4FCDD-1961-5BC7-B68A-E2565224E666

Fig. 5D, E

Sellanucheza jaegeri Golovatch 2013a: 20 (D, K).
Sellanucheza jaegeri – Golovatch 2013b: 330 (M, K); Nguyen and Sierwald 2013: 1296 (L).

##### Distribution.

China, Shaanxi Province, Taibai Shan, southern slope, above Houshenzi, 1,300–1,700 m a.s.l., 33°51'N, 107°50'E (Golovatch 2013a).

##### Remarks.

*Sellanucheza jaegeri* was described by Golovatch (2013a) from the Taibai Shan, Shaanxi Province, China. Somatically, the species is characterized by very poorly developed paraterga which are demarcated by sulci only dorsally. The gonopods are particularly distinctive, the femorite (fe) being suberect, slender and elongate. The solenophore (sph) is relatively short, strongly coiled and deeply bifid at tip (Fig. 5D, E). In addition, the base of the solenophore is supplied with two prominent processes, pa and pb, both being long, subunciform and directed distad (Fig. 5D, E). The species is currently known only from the type locality in Shaanxi Province, thus being endemic to northern China.

#### Sellanucheza typica

Taxon classificationAnimaliaPolydesmidaParadoxosomatidae

Golovatch, 2013

92493876-FB20-5948-8AA0-CC8ED9CF5F5B

Fig. 5F, G

Sellanucheza typica Golovatch 2013b: 329 (D, K).

##### Distribution.

China, Sichuan Province, Maoxian County, Southeast of Nanxinzhen, 3,155 m a.s.l., 31°34'04"N, 103°47'41"E (Golovatch 2013b).

##### Remarks.

This species shows poorly developed paraterga which are nonetheless traceable as bulges demarcated by sulci at least dorsally. It differs clearly from the structurally similar *S. variata* by the complete absence of pleurosternal carinae (vs present until ring 5 in *S. variata*). The gonopods are highly diagnostic, characterized by an elongate, coiled and slender femorite (fe) (Fig. 5F). The solenophore (sph) is subequal in length to the femorite, clearly coiled, and deeply bifurcated into an outer spiniform and an inner subspatuliform branch (Fig. 5F). The base of the solenophore bears a long, slender, acute process pa curved anteroventrad (Fig. 5F, G), while the distal part of the femorite is supplied with a short and spiniform process pb curved dorsad (Fig. 5G). These gonopodal features, particularly the slenderer femorite, make *S. typica* readily distinguished from *S. variata*, the latter possessing a significantly stouter femorite. The species is currently known only from the type locality (highest elevation of 3,155 m a.s.l. in this genus) in Sichuan Province, thus being endemic to northern China.

#### Sellanucheza laotica sp. nov.

Taxon classificationAnimaliaPolydesmidaParadoxosomatidae

35952EB1-2C88-5DF0-BC9E-2701C7004591

https://zoobank.org/4EFCED91-CE23-4AB7-ADDC-FDA6B1B6AA42

Figs 6, 7, 8

##### Type material.

• ***Holotype*** ♂ (CUMZ-11647), Laos, Khammouane Province, Nong Ping, Tham Nguen Mai, 230 m a.s.l., 17°22'14"N, 105°53'37"E, 24.02.2016, leg. J. Lips. • ***Paratypes*** 3 ♂, 4 ♀ (CUMZ-11647), same locality, together with holotype.

##### Diagnosis.

The new species is distinguished from all known congeners by the following combination of characters. Somatically, it differs from the Chinese *S. tenebra*, *S. typica* and *S. jaegeri* by the strongly developed paraterga on midbody rings (vs paraterga virtually absent or very poorly developed). From the large Vietnamese *S. grandis* and *S. hoffmani*, it is separated by the significantly smaller body size and the uniform, light yellowish to pallid coloration. A unique apomorphy seems to be the presence of well-developed ventroposterior tubercles on sterna near coxae, starting with ring 6. The gonopods are characteristic in the relatively short solenophore showing a truncate, subquadrate distal margin and an acute ventral angle. In addition, the species differs from *S. variata* and *S. jaegeri* by process pb being short, blunt and suberect (vs long, slender and acute or subunciform), and by the short, relatively stout solenomere which is subequal in length to the solenophore (vs long and flagelliform in other congeners).

##### Description.

Length 11.3–13.6 (♂) or 12.1–13.2 mm (♀), width of midbody pro- and metazona 0.8–1.2 and 1.2–1.4 mm (♂) or 0.9–1.2 and 1.2–1.4 mm (♀), respectively.

**Figure 6.**
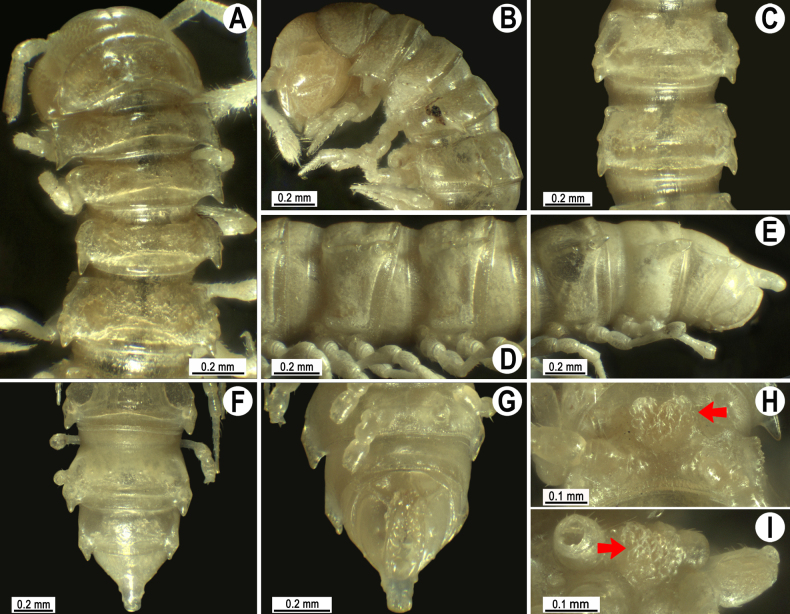
*Sellanucheza laotica* sp. nov., ♂ holotype. **A, B**. Anterior part of body, dorsal and lateral views, respectively; **C, D**. Rings 10 and 11, dorsal and lateral views, respectively; **E–G**. Posterior part of body, lateral, dorsal and ventral views, respectively; **H, I**. Sternal cones between coxae 4, subposterior and sublateral views, respectively. Red arrows indicate the position of the sternal cones.

Coloration of alcohol material after nine years of preservation uniform light yellowish to pallid; antennae and legs slightly paler (Fig. 6A–I).

Clypeolabral region and vertex sparsely setose, epicranial suture distinct. Antennae moderately long (Fig. 6A, B), reaching body ring 4 (♂) or 3 (♀) when stretched dorsally. In width, head < ring 3 < 4 < collum < ring 5 < 6 < 2 < 7–17 (♂, ♀) (Fig. 6A); thereafter body gently and gradually tapering. Collum with three transverse rows of setae: 4+4 anterior, 2+2 intermediate and 3+3 posterior; lateral margin with a small incision laterally in posterior half; posterior corner of paraterga very narrowly rounded, slightly declined ventrad, not extending past tergal margin (Fig. 6A, B).

Tegument smooth and shining (Fig. 6A–G), prozona very finely shagreened, metaterga smooth and leathery; surface below paraterga finely microgranulate (Fig. 6B, D, E). Postcollum metaterga with two transverse rows of setae: 3+3 in anterior (pre-sulcus) and 3+3 in posterior (post-sulcus) row. Tergal setae long, strong, slender, ca 1/3 metatergal length. Axial line faint, barely traceable on metaterga. Paraterga strongly developed (Fig. 6A–G), especially so in ♂, set at ca 1/3 midbody height, slightly upturned, but all lying below dorsum; shoulders well-developed, mostly rounded; posterior corner almost completely to very broadly rounded, all extending past tergal margin, posterior edge slightly concave, bent ventrad on rings 18 and 19 (Fig. 6F).

Paraterga 2 broad, anterior edge angular, lateral edge with two evident incisions in anterior half; posterior edge oblique (Fig. 6A, B). Lateral edges of paraterga 3 and 4 with two evident incisions: one in anterior 1/3, the other at midway (Fig. 6A). Following paraterga with three evident incisions: largest one in anterior 1/3, one at midway, and another near posterior corner (Fig. 6A, C). Paraterga thin blunt blades in lateral view, slightly thicker only on pore-bearing rings (Fig. 6B, D, E). Calluses on paraterga demarcated by sulci only dorsally. Ozopores evident, lateral, each lying in an ovoid groove at ca 1/4 in front of posterior corner (Fig. 6D, E). Transverse sulcus usually distinct, slightly incomplete on ring 4, complete and clearly visible on metaterga 5–18, usually narrow, shallow (Fig. 6A, C, F), superficial (due to coarse texture), not reaching bases of paraterga, slightly better developed in ♀. Stricture between pro- and metazona narrow, ribbed at bottom down to base of paraterga (Fig. 6A, C, F). Pleurosternal carinae complete crests, each usually with a sharp posterior tooth until ring 17, crests bulged anteriorly and with a small sharp posterior tooth each on rings 18 and 19 (♂, ♀) (Fig. 6B, D, E).

Epiproct (Fig. 6E–G) conical, rounded dorsoventrally, with two small apical papillae; tip subtruncate; pre-apical papillae evident, lying close to tip (Fig. 6F). Hypoproct roundly subtrapeziform (Fig. 6G), setiferous knobs at posterior edge well-separated and evident.

Sterna sparsely setose, cross-impressions shallow; starting on ring 6 with a pair of well-developed, ventroposterior tubercles near each coxa; a large, central, cordiform, densely setose lobe between ♂ coxae 4 (Fig. 6H, I). Legs moderately long and slender, midbody legs ca 1.0–1.3 (♂) or 0.9–1.2 × (♀) as long as body height, ♂ prefemora without modifications, tarsal brushes present until ♂ legs 4.

**Figure 7.**
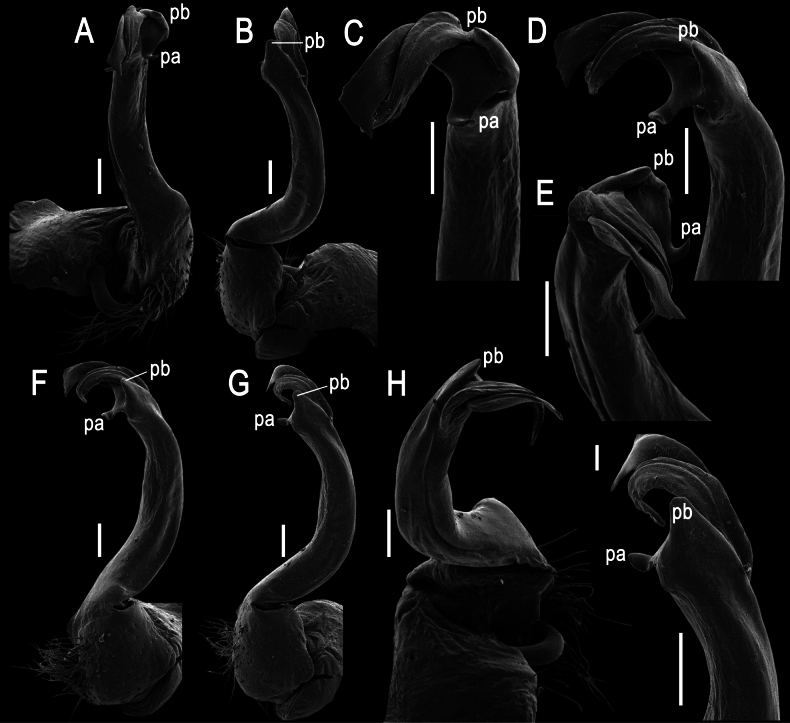
*Sellanucheza laotica* sp. nov., ♂ holotype, left gonopod. **A, B, F, G**. Submesal and lateral, anterior and subanterior views, respectively; **C–E, H, I**. Distal part, submesal, anterior, sublateral, subposterior and subanterior view respectively. Abbreviations: pa = process A, pb = process B. Scale bars: 0.05 mm.

**Figure 8.**
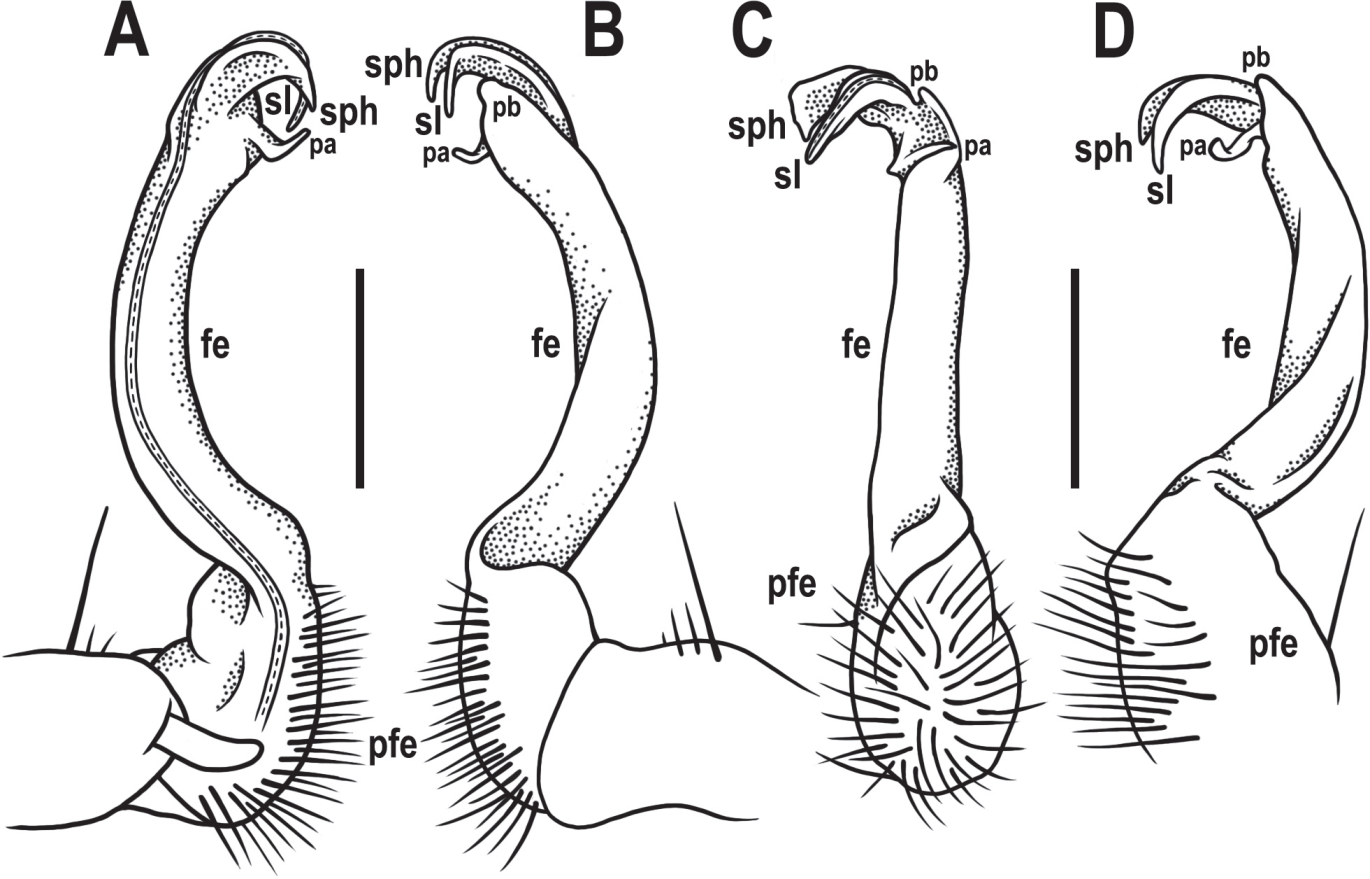
*Sellanucheza laotica* sp. nov., ♂ holotype, left gonopod. **A–F**. Mesal, lateral, anterior and subposterior views, respectively. Abbreviations: pa = process A, pb = process B, fe = femorite, pfe = prefemoral part, sl = solenomere, sph = solenophore. Scale bars: 0.1 mm.

Gonopods (Figs 7, 8) simple. Coxite long, slender, cylindrical, slightly curved posteriorly, densely setose distodorsally. Prefemorite (pfe) densely setose, as usual, ovoid, elongate, ca 1/3–1/4 length of acropodite (Figs 7A, 7B, 7F, 7G, 8A–C). Femorite (fe) long, slender, curved (Fig. 7B, F, G, 8A, B); distal part supplied with a short, blunt, suberect process pb (Figs 7B–I, 8B, 8C) and a short, slender, spiniform, slightly curved, mesal process pa (Figs 7A, 7C–G, 7I, 8A–D). Solenophore (sph) relatively short, supporting solenomere (sl), distal margin truncate, subquadrate, with an acute ventral angle (Figs 7C–F, 8C). Solenomere short, subequal in length to solenophore, relatively stout, tip acute and slightly projecting distad (Figs 7A, 7E–I, 8A–D).

##### Remarks.

*Sellanucheza laotica* sp. nov. represents the first record of the genus from a limestone cave in Khammouane Province, Laos. The distinct pallid coloration strongly suggests a troglobiomorphic adaptation to its subterranean existence, a characteristic frequently observed in other cave-dwelling millipedes within the region (Likhitrakarn et al. 2017, 2021, 2022, 2024; Liu and Wynne 2019; Golovatch and Liu 2020). The new species appears to be narrowly endemic to its karst cave systems in northeastern Laos.

##### Etymology.

The specific epithet is a Latin adjective referring to Laos, the country of origin where the type material was collected.

#### Sellanucheza longispina sp. nov.

Taxon classificationAnimaliaPolydesmidaParadoxosomatidae

4A258ECA-2C05-5A2D-9CE2-B31EF528EBEF

https://zoobank.org/A5DD1D34-5D27-404F-A44E-C466D57007E8

Figs 9, 10, 11

##### Type material.

• ***Holotype*** ♂ (SMF), Laos, Bolikhamsay Province, Lak Sao, Tham Mankhone, large halled tourist cave (Fig. 15A, B), with some electric lights at the entrance, 501 m a.s.l., 18°13'16.1"N, 104°48'45.9"E, 21.07.2016, leg. P. Jäger.

##### Diagnosis.

The new species is distinguished from congeners by its large size (ca 49 mm) and the unique presence of sternal cones on both rings 5 and 6 (vs usually either ring 5 alone or ventroposterior tubercles from ring 6 on in *S. laotica* sp. nov.). It also differs from the pallid *S. laotica* sp. nov. by its dark blackish brown coloration. The gonopods are characteristic: process A (pa) long, spiniform and subequal in length to the solenophore (vs shorter, spiniform and suberect in *S. laotica* sp. nov., or the solenophore much shorter in *S. grandis*); process B (pb) large, laminiform and bifid (vs small and acute in *S. variata* and *S. grandis*); the solenophore is stout and deeply bifid, while the solenomere is flagelliform (vs stout in *S. laotica* sp. nov.).

##### Description.

Length 48.6 mm (♂), width of midbody pro- and metazona 4.8 and 5.7 mm (♂), respectively.

**Figure 9.**
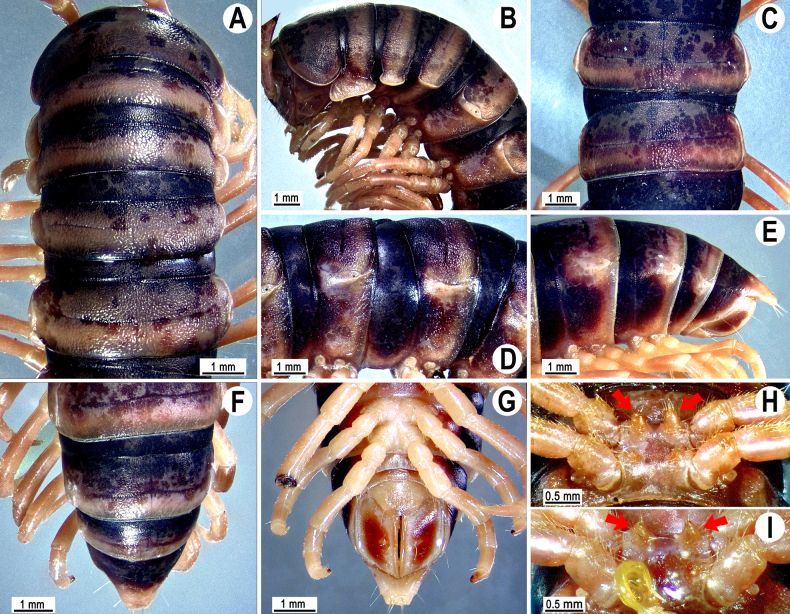
*Sellanucheza longispina* sp. nov., ♂ holotype. **A, B**. Anterior part of body, dorsal and lateral views, respectively; **C, D**. Rings 10 and 11, dorsal and lateral views, respectively; **E–G**. Posterior part of body, lateral, dorsal and ventral views, respectively; **H, I**. Sternal cones on rings 5 and 6, subposterior views, respectively. Red arrows indicate the position of the sternal cones.

Coloration of alcohol material after nine years of preservation, faded to blackish brown (Fig. 9B–H), posterior halves of metaterga, paraterga and epiproct pale light brown to yellowish (Fig. 9A–F), head and antennae dark brown, tip of antenna dark brown, venter and legs pale brown to yellowish (Fig. 9G–I).

Clypeolabral region densely setose (Fig. 9B), vertex sparsely so, epicranial suture distinct. Antennae moderately long, reaching body ring 4 (♂) when stretched dorsally. In width, head < ring 4 < 3 < 5 < 6 < 7 < collum < ring 2 < 8 < 9–17 (♂) (Fig. 9A); thereafter body gently and gradually tapering.

Collum semilunar, setation pattern untraceable; surface rugulose, middle slightly flattened; paraterga rounded and subtriangular, devoid of lateral incisions; posterior corner very narrowly rounded, not extending past tergal margin (Fig. 9A, B).

Tegument smooth and shining, prozona finely shagreened, metaterga smooth and leathery; surface below paraterga finely microgranulate (Fig. 9A–G). Postcollum metaterga with two transverse rows of setae traceable at least as insertion points: 3+3 in anterior (pre-sulcus) and 4+4 in posterior (post-sulcus) row; posterior row barely traceable. Axial line visible both on pro- and metazona.

Paraterga well developed (Fig. 9A–E, F), set high (at upper 1/3 of body), slightly upturned, but lying below dorsum; anterior edge broadly rounded and narrowly bordered, fused to callus; lateral edge without incisions; posterior corner very narrowly rounded, not produced past rear tergal margin except for rings 2 and 3 (Fig. 9A, B); posterior edge nearly straight. Paraterga 2 broad, anterior edge angular and rounded, lateral edge without incisions (Fig. 9A). Calluses on paraterga narrow, demarcated by a sulcus only dorsally. Ozopore evident, lateral, lying in an ovoid groove at ca 1/3 metatergal length in front of posterior corner (Fig. 9B, D, E).

Transverse sulcus distinct (Fig. 9A, C, F), complete on metaterga 5–18, narrow, line-shaped, deep, reaching bases of paraterga, faintly ribbed at bottom, incomplete and nearly wanting on rings 4 and 19 (Fig. 9A, F). Stricture between pro- and metazona narrow, ribbed at bottom down to base of paraterga (Fig. 9C, D). Pleurosternal carinae complete high crests, each with a sharp posterior tooth on rings 2–4, thereafter increasingly strongly reduced and remaining visible only as a bulge anteriorly until ring 7 (Fig. 9B, D, E).

Epiproct (Fig. 9E–G) conical, rounded dorsoventrally, with two small apical papillae; tip subtruncate; pre-apical papillae evident, lying close to tip (Fig. 9G). Hypoproct nearly semi-circular (Fig. 9G), posterior tip broadly rounded, setiferous knobs at posterior edge very small and moderately well separated.

Sterna sparsely setose, without modifications; cross-impressions shallow (Fig. 9G). Rings 5 and 6 each with a paramedian pair of evident, basally contiguous cones located anteriorly near each coxa; cones between coxae 4 and 6 being more prominent and larger than those between coxae 5 and 7 (Fig. 9H, I). A paramedian pair of small, but evident tubercles in front of gonopod aperture. Legs moderately long and slender, midbody legs ca 1.1–1.3 × as long as body height; ♂ prefemora without modifications; tarsal brushes present until ♂ ring 12.

**Figure 10.**
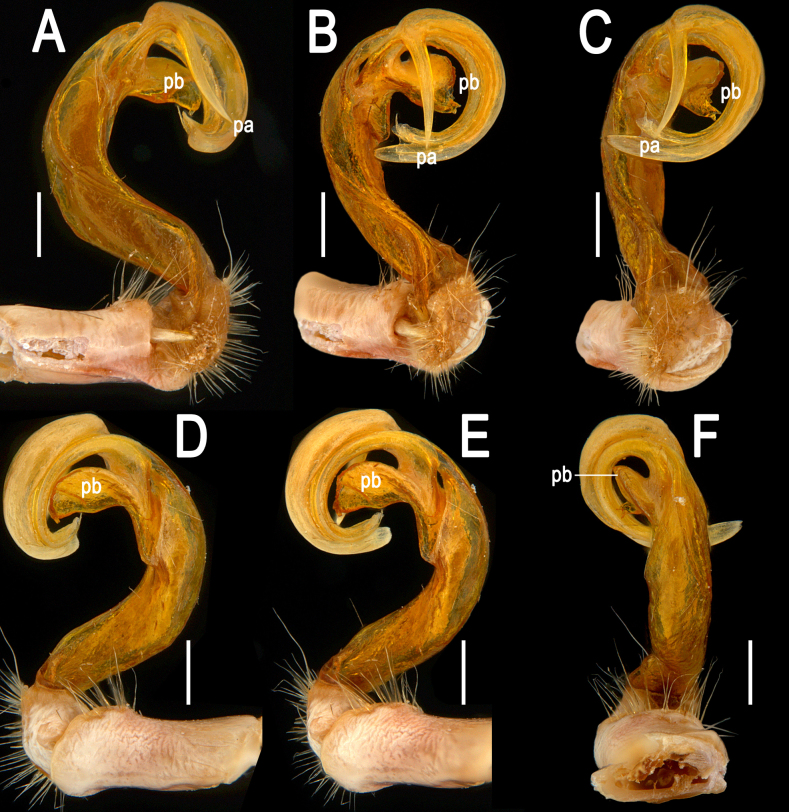
*Sellanucheza longispina* sp. nov., ♂ holotype, left gonopod. **A–F**. Mesal, submesal, subanterior, lateral, sublateral and posterior views, respectively. Abbreviations: pa = process A, pb = process B. Scale bars: 0.2 mm.

**Figure 11.**
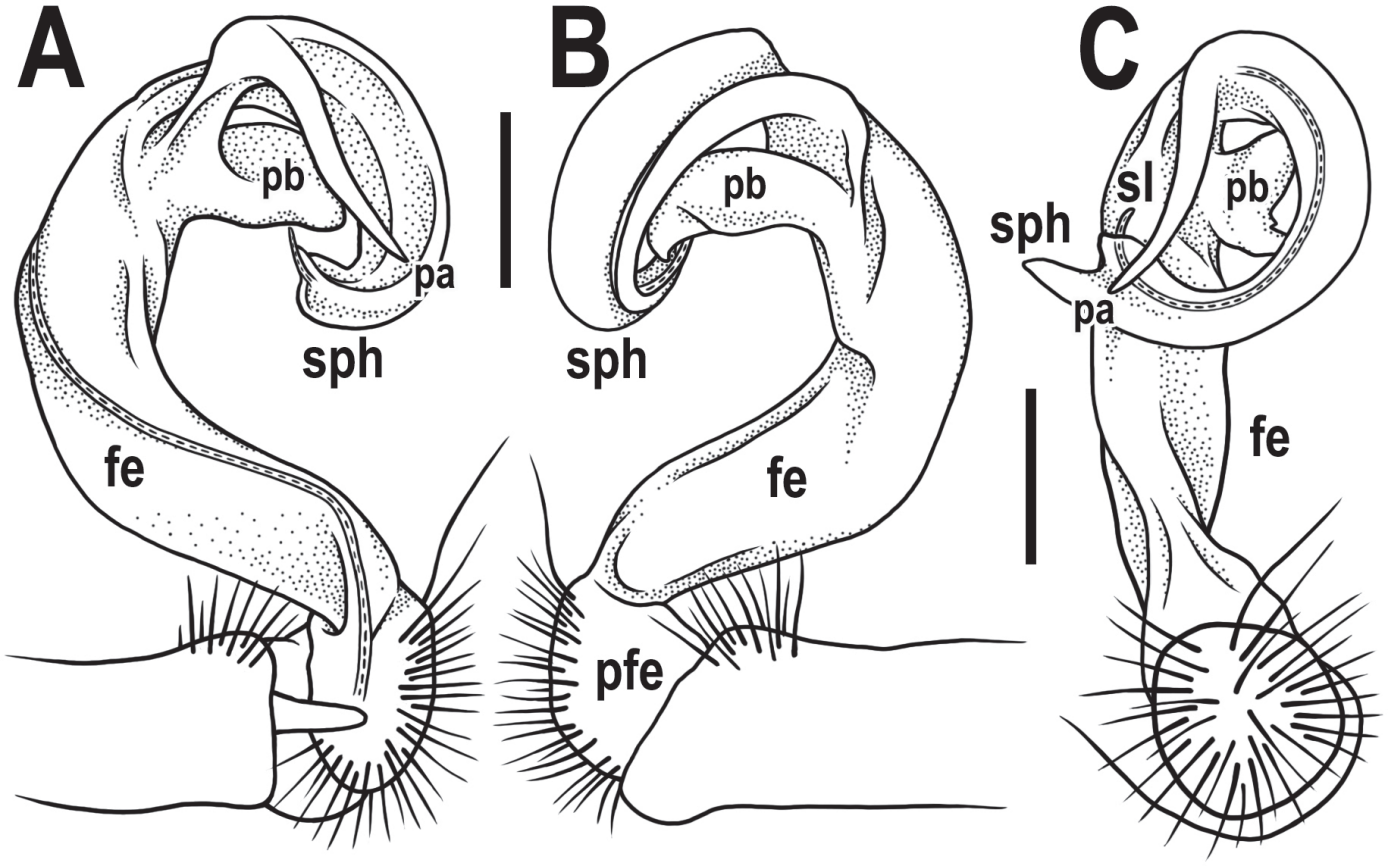
*Sellanucheza longispina* sp. nov., ♂ holotype, left gonopod. **A–F**. Mesal, submesal, subanterior, lateral, anterior and posterior views, respectively. Abbreviations: pa = process A, pb = process B, fe = femorite, pfe = prefemoral part, sl = solenomere, sph = solenophore. Scale bars: 0.5 mm.

Gonopods (Figs 10, 11) relatively simple, clearly coiled. Coxite long, slender, cylindrical, suberect, densely setose distodorsally (Figs 10A, 10D, 11A, 11B). Prefemorite (pfe) densely setose, as usual, ovoid, elongate, short, ca 1/4–1/5 length of acropodite (Figs 10A–E, 11A–C). Femorite (fe) relatively stout, long, curved dorsad (Figs 10A, 10B, 10D, 10E, 11A, 11B); distal part supplied with a prominent, stout, laminiform, linguiform, apically rounded process pb directed anteriad, the latter being bifid (Figs 10A–F, 11A–C), with ventral lobule further split into two small acute points (Figs 10A–C, 10F, 11C). Process pa conspicuous, spiniform, long, slender, acute, subequal in length to solenophore, curved anteromesad (Figs 10A–C, 11A, 11C). Solenophore (sph) long, stout, strongly coiled, deeply bifid distally, both lobules being blunt or rounded (Figs 10A–E, 11A, 11C); solenophore almost completely sheathing solenomere. Solenomere (sl) long, slender, flagelliform, tip acute and slightly projecting distad past solenophore (Figs 10A–E, 11A, 11C).

##### Remarks.

*Sellanucheza longispina* sp. nov. represents a second species of the genus to be recorded from a limestone cave environment in northeastern Laos, specifically marking the first record from Bolikhamsay Province. Compared to the pallid *S. laotica* sp. nov., this species retains dark pigmentation, suggesting that it may be a troglophile. Morphologically, the presence of sternal cones on both rings 5 and 6 represents a unique novelty for the genus. Geographically, the species appears to be narrowly endemic to the karst systems of Bolikhamsay Province it inhabits.

##### Etymology.

To emphasize the distinctively long and spiniform gonopodal process A; noun in apposition.

#### Sellanucheza ancorata sp. nov.

Taxon classificationAnimaliaPolydesmidaParadoxosomatidae

321A5CF6-7F3B-50B6-91C9-B4BFC9D1541B

https://zoobank.org/22CECC4A-5229-4BEE-8281-42A9DE0D65C3

Figs 11, 12, 13

##### Type material.

• ***Holotype*** ♂ (SM-08), Laos, Khammouane Province, 2.5 km west-northwest of Ban Tathot, Tham Kamouk, large halled limestone cave, 200 m a.s.l., 17°37'53.5"N, 105°07'25.7"E, 06.08.2016, leg. P. Jäger.

##### Diagnosis.

The new species is distinguished from all congeners by the unique, anchor-shaped (trifid) solenophore tip. Somatically, it differs from the two other Laotian new species above by its intermediate size (ca 21 mm) and uniform brown yellowish coloration (vs small and pallid in *S. laotica* sp. nov.; large and dark in *S. longispina* sp. nov., lacking the specific sternal modifications found in either species). The gonopods (Figs 13, 14) are highly characteristic: process pb short, stout and laminiform (vs large, laminiform and bifid in *S. longispina* sp. nov., long and subunciform in *S. jaegeri*, or small and acute in *S. tenebra*); process pa distinctively long, slender and spiniform with an acute tip directed ventromesad (vs shorter, spiniform and suberect in *S. laotica* sp. nov., or large and acute in *S. grandis*) and the solenophore tip is split into three distinct prongs (central straight, lateral recurved), a characteristic unique to this species.

##### Description.

Length 20.8 mm (♂), width of midbody pro- and metazona 1.6 and 2.1 mm (♂), respectively.

**Figure 12.**
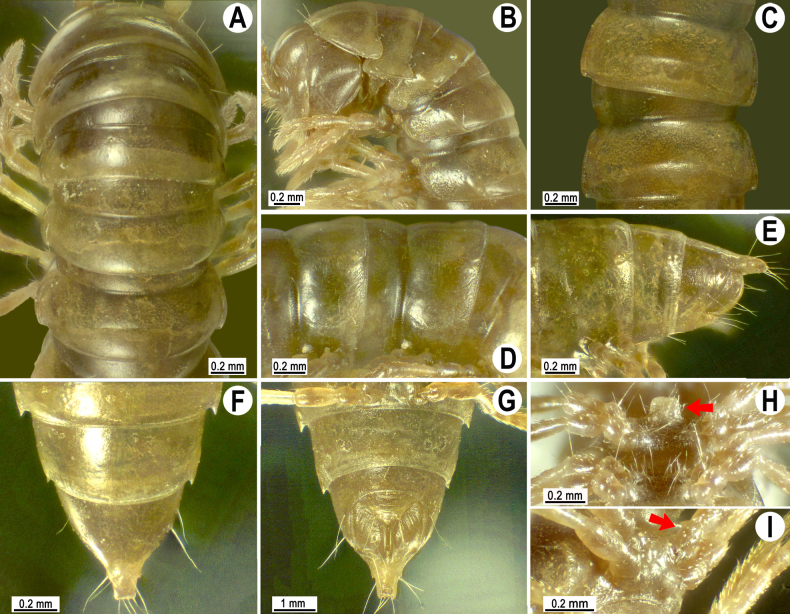
*Sellanucheza ancorata* sp. nov., ♂ holotype. **A, B**. Anterior part of body, dorsal and lateral views, respectively; **C, D**. Rings 10 and 11, dorsal and lateral views, respectively; **E–G**. Posterior part of body, lateral, dorsal and ventral views, respectively; **H, I**. Sternal cones between coxae 4, subposterior and sublateral views, respectively. Red arrows indicate the position of the sternal cones.

Coloration of alcohol material after nine years of preservation uniform brown yellowish (Fig. 12A–G); legs and antennae a little paler, tip of antenna dark brown.

Clypeolabral region and vertex sparsely setose, epicranial suture distinct. Antennae moderately long, reaching body ring 3 (♂) when stretched dorsally. In width, head < ring 3 < 4 < collum < ring 2 < 5 < 6 < 7–17 (♂); thereafter body gently and gradually tapering.

Collum with three transverse rows of setae: 4+4 anterior, 2+2 intermediate and 3+3 posterior; lateral margin with a small incision in posterior half; posterior corner of paraterga very narrowly rounded, slightly declined ventrad, extending past tergal margin (Fig. 12A, B).

Tegument smooth and shining, prozona very finely shagreened, metaterga smooth and faintly rugulose (Fig. 12A–G); surface below paraterga finely microgranulate (Fig. 12B, D, E). Postcollum metaterga with two transverse rows of setae: 2+2 in anterior (pre-sulcus) and 3+3 in posterior (post-sulcus) row. Tergal setae long, strong, slender, ca 1/3 metatergal length. Axial line faint, barely traceable on metaterga.

Paraterga well developed (Fig. 12A, C, F), set high (at upper 1/3 of body), subhorizontal, lying below dorsum; anterior edge broadly rounded and narrowly bordered, fused to callus; lateral edge without incisions; posterior corner very narrowly rounded, not extending past tergal margin except for rings 17–19 where it becomes increasingly pointed (Fig. 12E, F); posterior edge nearly straight. Paraterga 2 broad, slightly upturned posteriorly, anterior edge rounded, lateral edge with a small incision in anterior half; posterior edge concave (Fig. 12B). Calluses on paraterga narrow, each demarcated by a sulcus only dorsally. Ozopores evident, lateral, each lying in an ovoid groove at ca 1/4 metatergal length in front of posterior corner (Fig. 12B, D, E).

Transverse sulci distinct (Fig. 12A, C, F), complete on metaterga 6–18, narrow, line-shaped, shallow, not reaching the bases of paraterga, smooth at bottom, incomplete and nearly wanting on ring 5. Stricture between pro- and metazona narrow, beaded at bottom down to base of paraterga (Fig. 12A, C). Pleurosternal carinae complete crests on anterior rings, thereafter gradually reduced posteriad until ring 16 (♂) (Fig. 12B, D, E).

Epiproct (Fig. 12E–G) conical, rounded dorsoventrally, with two small and evident apical papillae; tip subtruncate; pre-apical papillae small, lying close to tip (Fig. 12F, G). Hypoproct roundly subtriangular (Fig. 12G), setiferous knobs at posterior edge well-separated and evident.

Sterna sparsely setose, cross-impressions shallow, without modifications; a single, linguiform, densely setose, sternal lobe between ♂ coxae 4 (Fig. 12H, I). Legs moderately long and slender, midbody legs ca 1.1–1.3 × as long as body height; ♂ prefemora without modifications; tarsal brushes present until ♂ ring 16.

**Figure 13.**
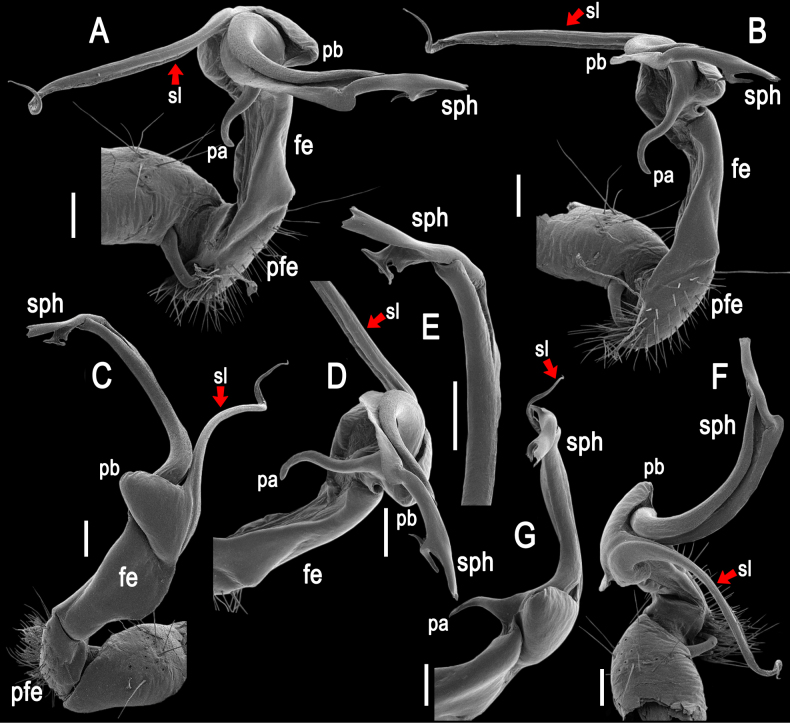
*Sellanucheza ancorata* sp. nov., ♂ holotype, left gonopod. **A–C, F**. Mesal, submesal, lateral and subposterior views, respectively; **D, E, G**. Distal part, submesal, lateral and subanterior views, respectively. Abbreviations: fe = femorite, pa = pa, pb = pb, pfe = prefemoral part, sl = solenomere, sph = solenophore. Red arrows with “sl” labels indicate the solenomere, which is dislodged from its natural position (solenophore sheath) due to a drying artifact in most views. Note that in view **G**, the solenomere (sl) remains in its natural position, fully sheathed by the solenophore (sph) (see also Fig. 14 for the natural structure). Scale bars: 0.1 mm.

**Figure 14.**
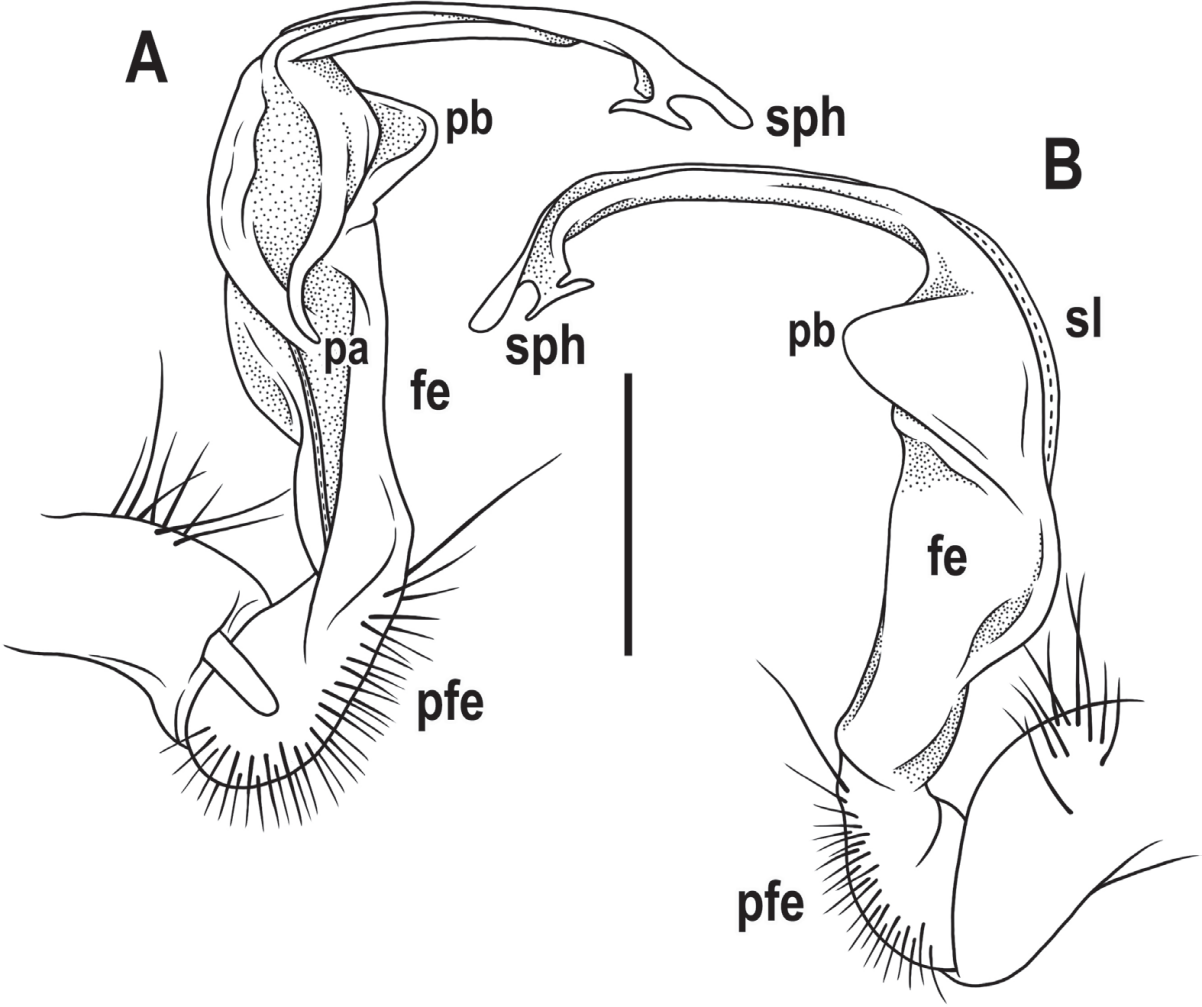
*Sellanucheza ancorata* sp. nov., ♂ holotype, left gonopod. **A, B**. Mesal and lateral views, respectively. Abbreviations: pa = process A, pb = process B, fe = femorite, pfe = prefemoral part, sl = solenomere, sph = solenophore. Scale bars: 0.2 mm.

Gonopods (Figs 13, 14) simple. Coxite long, stout, subcylindrical, relatively straight, but slightly curved dorsally, with a dense group of setae distodorsally (Figs 13A, 13B, 13C, 14A, 14B). Prefemorite (pfe) densely setose, as usual, relatively short, ca 1/3 length of acropodite (Figs 13A, 13B, 13C, 14A, 14B). Femorite (fe) stout, relatively long, suberect, with a distinct. clearly visible lateral suture (Figs 13A–D, 14A, 14B). Distal part of femorite supplied with two distinct processes: process pb (pb) short, stout, laminiform, elevated dorsally, with a rounded apical margin (Figs 13A–C, 13G, 13F, 14A, 14B); process pa distinctively long, slender, spiniform, clearly curved, with an acute tip directed ventromesad (Figs 13A, 13B, 13D, 13G, 14A). Solenophore (sph) long, slender, directed anteriad and slightly curved; tip highly characteristic, trifid; central prong straight to slightly curved, while two lateral processes recurved and pointing basally (Figs 13A–G, 14A, 14B). Solenomere long, slender, almost entirely sheathed by solenophore, forming a distinct anchor shape (Figs 13G, 14A, 14B).

##### Remarks.

**Figure 15.**
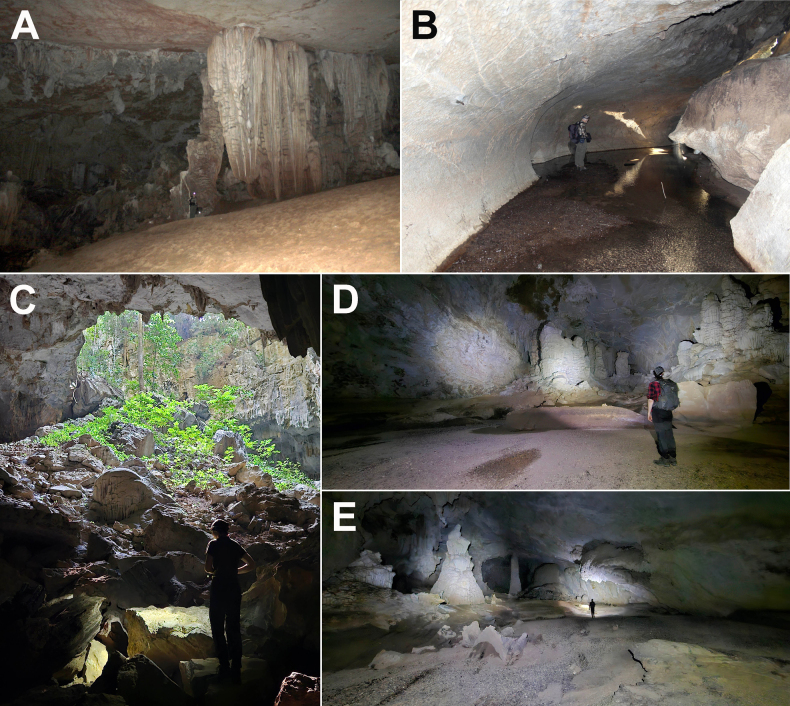
Habitats of new *Sellanucheza* species from Laos. **A, B**. Tham Mankhone cave (type locality of *S. longispina*); **A**. General view of the main hall, with large parts covered with sand; **B**. The end of the cave showing a high-up opening (window) connecting to the surrounding jungle; **C, D**. Tham Kamouk cave (type locality of *S. Ancorata*); **C**. The end of the cave showing a ceiling breakdown with massive litter accumulation from the forest above; **D**. General view of the large-halled limestone cave. Photographs by P. Jäger.

It seems noteworthy that the natural structure of the gonopod is clearly shown in Fig. 14, 13G. The structure depicted in most other views of Fig. 13 exhibits a distortion caused by the drying of the specimen during preparation. This process caused the solenomere (sl) to dislodge from the solenophore sheath and project posteriorly, an artifact that does not reflect the true morphology of the species. Geographically, *Sellanucheza ancorata* sp. nov. is currently known only from the type locality in Khammouane Province. This is a large-halled limestone cave (Fig. 15D, E) which is not visited by tourists, although it is occasionally used by villagers as a shortcut between villages. The end of the cave features a ceiling breakdown with massive litter accumulation from the forest above (Fig. 15C). In the rainy season, a river flows through the cave, and bats are present. The species appears to be narrowly endemic to its limestone karst system of the area, a pattern characteristic of many members of this genus in the Indochinese Peninsula.

##### Etymology.

To emphasize the characteristic anchor-shaped (trifid) tip of the solenophore; adjective.

### Key to species of *Sellanucheza* (based mainly on adult males)

**Table:** 

1	Paraterga 3–19 present, but strongly reduced, represented only by faint bulges and not delimited by a sulcus even dorsally. Gonopodal solenophore (sph) with two large, rounded, subequal lobes at apex (Fig. 1A, B). Sichuan, China	***S. tenebra* (Hoffman, 1961)**
–	Paraterga present, ranging from poorly to strongly developed, always delimited by a sulcus at least dorsally. Gonopodal solenophore (sph) structures otherwise	**2**
2	Gonopodal solenophore (sph) short and stout, subequal in length to solenomere, height approx. 1/3–1/4 of gonopodal femorite height	**3**
–	Gonopodal solenophore (sph) long and strongly coiled, extending past 1/3 of gonopodal femorite height; solenomere long and flagelliform	**5**
3	Body small (length 11.3–13.6 mm, width 1.2–1.4 mm), pallid (unpigmented); paraterga strongly developed (Fig. 6A–G), sterna near coxae with well-developed ventroposterior tubercles starting with ring 6 on	***S. laotica* sp. nov**.
–	Body larger (length > 20 mm, width > 2.7 mm), paraterga very poorly developed (Figs 2A–G, 9A–F, 12A–G); pigmented (brown to blackish) (Figs 2A–F, 9A–F, 12A–F); sterna without continuous ventroposterior tubercles. Vietnam or China	**4**
4	Body smaller (length 30–33 mm, width 2.7–3.4 mm); male sternum 4 with a single, linguiform, densely setose lobe. Base of solenophore (sph) with two processes, pa and pb, suberect, long and subunciform; solenophore tip clearly bifid (Fig. 5E, D). Shaanxi Province, China	***S. jaegeri* Golovatch, 2013**
–	Body larger (length 54.3–57.3 mm, width 5.7–7.0 mm). male sternum 4 with a large, central, slightly bifid lobe (Fig. 4D). Base of solenophore (sph) with two processes, pa and pb, both acute and directed ventrolaterad, and supplied with an additional small, dentiform, sublateral spine (d) (Fig. 4A, B), solenophore tip only faintly bifid (Fig. 4A, C). Ninh Binh, Vietnam	***S. grandis* (Golovatch, 1984)**
5	Gonopodal Solenophore (sph) long, slender, directed anteriad and slightly curved; tip trifid, with two lateral processes recurved and pointing basally to form an anchor shape (Figs 13A–G, 14A, 14B); process pa distinctively long, slender, spiniform and directed ventrad (Figs 13A, 13B, 13D, 13G, 14A); process pb short, stout and laminiform (Figs 13A–C, 13G, 13F, 14A, 14B). Khammouane, Laos	***S. ancorata* sp. nov**.
–	Gonopodal solenophore (sph) evidently coiled; tip not anchor-shaped (simple, bifid or truncate) (Figs 3B, 3C, 5A–C, 5G, 5F, 10A–E, 11A, 11C). Process pb otherwise (Figs 3A, 3C, 5A–C, 5G, 10A–F, 11A–C)	**6**
6	Sterna of both rings 5 and 6 each with a paramedian pair of evident, basally contiguous cones located anteriorly near each coxa (Fig. 9H, I). Gonopod process pb large, laminiform and evidently bifid (Figs 10A–F, 11A–C). Bolikhamsay, Laos	***S. longispina* sp. nov**.
–	Sterna of only ring 5 modified (Fig. 2H, I). Gonopod process pb spini- or dentiform (Figs 3A, 3C, 5A–C, 5G)	**7**
7	Body larger (length 52–59 mm, width 5.3–6.4 mm). Gonopodal solenophore (sph) at least ca half as long as gonopodal femorite (fe) (Fig. 5A, B). Process pb long, basal, laminiform (Fig. 5A–C). Process pa very long, nearly straight, spiniform (Fig. 5A, C). Kon Tum, Vietnam	***S. hoffmani* Nguyen, 2011**
–	Body smaller (length 37–46 mm, width 3.3–5.0 mm). Gonopodal solenophore (sph) clearly curved and subequal in length to gonopodal femorite (fe) (Figs 3A–C, 5F). Process B (pb) short, spiniform, slightly curved upwards (Figs 3A, 3C, 5G). Process A (pa) long, clearly curved and acute (Figs 3A–C, 5F, 5G)	**8**
8	Pleurosternal carinae absent. Male legs long, ca 1.7–1.8 × as long as body height; tarsal brushes present until body rings 12. Gonopodal femorite (fe) elongate and slender (Fig. 5F). Sichuan, China	***S. typica* Golovatch, 2013**
–	Pleurosternal carinae present until ring 5 (Fig. 2B, D, E). Male legs moderately long, ca 1.2–1.4 × as long as body height; tarsal brushes present until body rings 16. Gonopodal femorite (fe) stout (Fig. 3A–C). Lao Cai, Vietnam	***S. variata* (Attems, 1953)**

## Discussion

**Figure 16.**
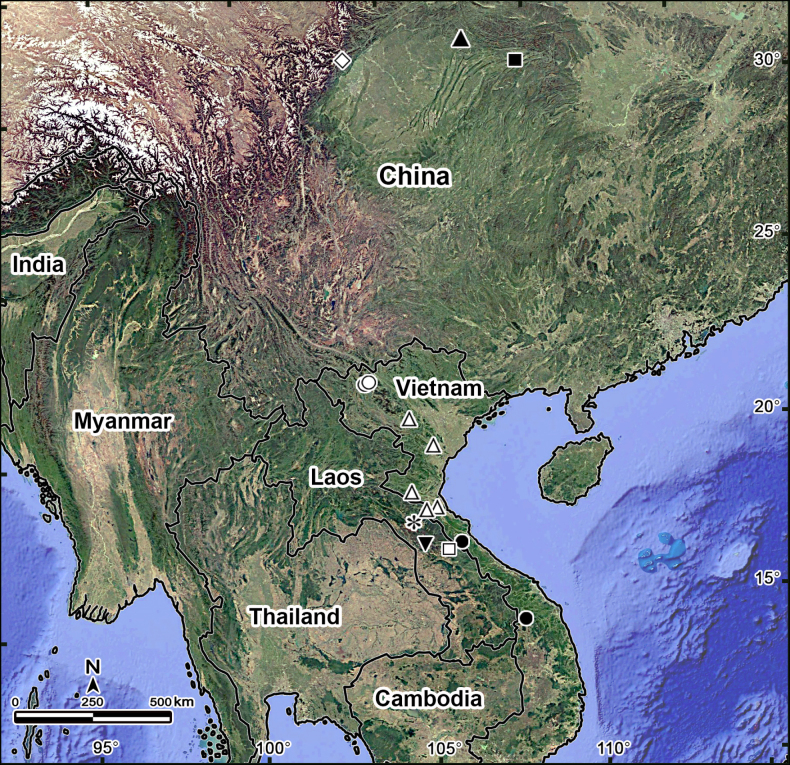
Distribution of all currently known *Sellanucheza* (nine species) Filled triangle *S. jaegeri* Golovatch, 2013 Open diamond *S. typica* Golovatch, 2013 Filled square *S. tenebra* (Hoffman, 1961) Open circle *S. variata* (Attems, 1953) Open triangle *S. grandis* (Golovatch, 1984) Asterisk *S. longispina* sp. nov. Inverted filled triangle *S. ancorata* sp. nov. Open square *S. laotica* sp. nov. Filled circle *S. hoffmani* Nguyen, 2011.

The genus *Sellanucheza* has hitherto been considered as being restricted to southern China (*S. tenebra*, *S. jaegeri*, and *S. typica*) and northern to central Vietnam (*S. variata*, *S. grandis*, and *S. hoffmani*) (Hoffman 1961; Golovatch 1984, 2013a, 2013b; Nguyen 2011). The present discovery of *S. laotica* sp. nov., *S. longispina* sp. nov., and *S. ancorata* sp. nov. in the limestone karst caves of Khammouane and Bolikhamsay provinces represents the first formal records of the genus from Laos (Fig. 16). These findings are of particular zoogeographical significance as they represent a major range extension of the genus from its previously known limits in China and Vietnam into the interior of the Indochinese Peninsula (Laos). This discovery underscores the wider distribution of the genus across the region than previously documented.

With the addition of the three species described above, the known millipede fauna of Laos has expanded to 99 accepted species belonging to 34 genera, 12 families, and eight orders. This fauna is heavily dominated by the order Polydesmida (57 species), followed by Spirostreptida (13 species) and Sphaerotheriida (12 species); together, these three orders account for approximately 83% of the total richness reported (e.g., Likhitrakarn et al. 2014a, 2017, 2023b, 2024, 2025a, 2025b, 2026; Srisonchai et al. 2023a, 2023b; Srikampha et al. 2025). As is characteristic of the Indochinese region, the family Paradoxosomatidae remains the most speciose group, currently represented in Laos by 46 species in 13 genera, accounting for over 46% of the total diversity. The steady accumulation of records in *Sellanucheza*, alongside other diverse genera such as *Tylopus* (15 species) (Srisonchai et al. 2023b; Likhitrakarn et al. 2025a), *Sphaerobelum* (11 species) (Srikampha et al. 2025), and *Orthomorpha* (8 species) (Likhitrakarn et al. 2014b), clearly demonstrates that the actual diversity of the Laotian diplopod fauna is significantly higher than originally documented, likely exceeding 130 species (Likhitrakarn et al. 2017). Continued exploration of the vast, unsampled karst areas of the Annamite Range is essential to adequately document the unique biodiversity of this region.

## Supplementary Material

XML Treatment for Sellanucheza

XML Treatment for Sellanucheza tenebra

XML Treatment for Sellanucheza variata

XML Treatment for Sellanucheza grandis

XML Treatment for Sellanucheza hoffmani

XML Treatment for Sellanucheza jaegeri

XML Treatment for Sellanucheza typica

XML Treatment for Sellanucheza laotica

XML Treatment for Sellanucheza longispina

XML Treatment for Sellanucheza ancorata

## Additional information

### Artificial Intelligence (AI) use

During the preparation of this manuscript, the authors used AI tools solely for English grammar correction and language polishing. After using these services, the authors reviewed and edited the content as needed and take full responsibility for the final content of the manuscript.

### Author ORCIDs

Natdanai Likhitrakarn https://orcid.org/0000-0002-1306-317X

Sergei I. Golovatch https://orcid.org/0000-0001-7159-5484

Josiane Lips https://orcid.org/0009-0004-8403-5506

Somsak Panha https://orcid.org/0000-0002-4431-2458

Chirasak Sutcharit https://orcid.org/0000-0001-7670-9540

### Data availability

All of the data that support the findings of this study are available in the main text.
